# Late Thrombosis of Sirolimus-Eluting Stent: A Multifactorial Problem

**DOI:** 10.1155/2009/816715

**Published:** 2009-11-04

**Authors:** Igor Kranjec, Andreja Cerne

**Affiliations:** Department of Cardiology, University Medical Centre Ljubljana, 1000 Ljubljana, Slovenia

## Abstract

We report a case of a young patient in whom a sirolimus-eluting stent was implanted on the culprit left anterior descending coronary artery at primary percutaneous coronary intervention (PCI) for acute myocardial infarction. Nine months later she suffered from a reinfarction due to the late stent thrombosis despite a continuous antiplatelet therapy with aspirin and clopidogrel. A cluster of factors that might have contributed to the development of the stent thrombosis were identified: suboptimal PCI technique, complete stent fracture, and clopidogrel resistance. The obstructed stent was successfully reopened by repeat PCI, while the clopidogrel maintenance dosage was doubled to 150 mg daily for the following year. The further long-term clinical course was uneventful.

## 1. Introduction

Predictors of the drug-eluting stent (DES) thrombosis (ST) fall usually into four categories: (1) procedural factors (e.g., incomplete stent expansion), (2) drug effects (e.g., delayed vessel wall healing), (3) DES platform (e.g., hypersensitivity to the polymer), and (4) antiplatelet activity (e.g., clopidogrel resistance). Recent investigation of sirolimus-eluting stents (SES) using optical coherence tomography discovered that only 16% of all stent struts were completely endothelialized at 6-month follow-up. Moreover, distinct thrombi appeared in some of the SESs though ie of the patients suffered any harmful consequences [1]. It was concluded that additional mechanisms along with thrombogenic struts would be needed to trigger the ST.

We describe, thus, a case of the young patient in whom the SES was implanted on the culprit LAD artery at primary PCI for the acute myocardial infarction (MI). A cluster of unfavorable factors resulted in the late ST despite a continuous double antiplatelet therapy. A repeat PCI was chosen to open the clogged stent with a good long-term result.

## 2. Case Report

**Figure 1:**
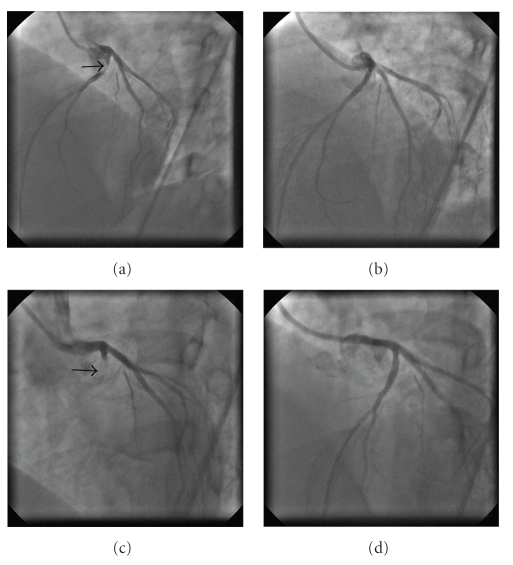
Consecutive coronary angiograms are shown in the LAO view. (a) Tight, thrombotic lesion (arrow) of the angulated part of the LAD artery. (b) Good angiographic result is achieved after implantation of a 2.5 × 18 mm sirolimus-eluting stent. (c) Thrombotic occlusion (arrow) 9 months after the stent implantation. (d) Final angiographic result after successive inflations of a 3.0 × 20 mm noncompliant balloon.

A 38-year-old female with hypertension, hypercholesterolemia, and positive family history presented to the ED one hour after the onset of a severe chest discomfort. Her ECG showed marked ST-segment elevations in leads I, aVL, V_1–4_. She was taken immediately to the catheterization laboratory after being given nitroglycerin, morphine, aspirin, and UFH. Angiography revealed a tight thrombotic lesion of the proximal LAD with a TIMI grade 2 blood flow (Figure 1(a)). After additional ACT-guided UFH and 600 mg of clopidogrel, a JL 3.5 F6 guiding catheter (Cordis, Miami, Fla, USA) was used to intubate the LCA, a 0.014-inch Asahi soft guide wire (Abbot, Redwood City, Calif, USA) was introduced into the distal LAD, a 2.0 × 20 mm Mercury balloon (Abbot, Redwood City, Calif, USA) was inflated once at 7 At to pre-dilate the lesion, and finally a 2.5 × 18 mm SES (Cypher, Cordis, Miami, FL, USA) was implanted at 16 At on the proximal LAD. The angiographic control showed a good result (Figure 1(b)) and, as a result, no attempt was made to expand the stent with a noncompliant balloon.

**Figure 2:**
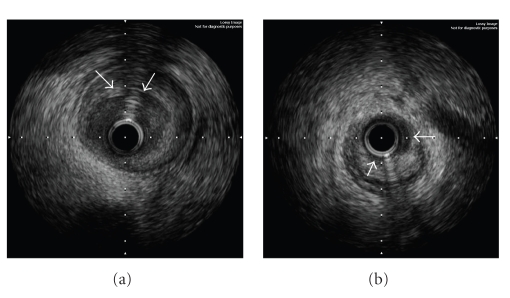
IVUS images of the LAD artery beyond the proximal (a) and distal borders (b) of the stented segment are shown after the occlusive thrombus was mechanically removed. A large and predominantly fibrous plaque (arrows) narrows the vessel lumen particularly at distal site.

**Figure 3:**
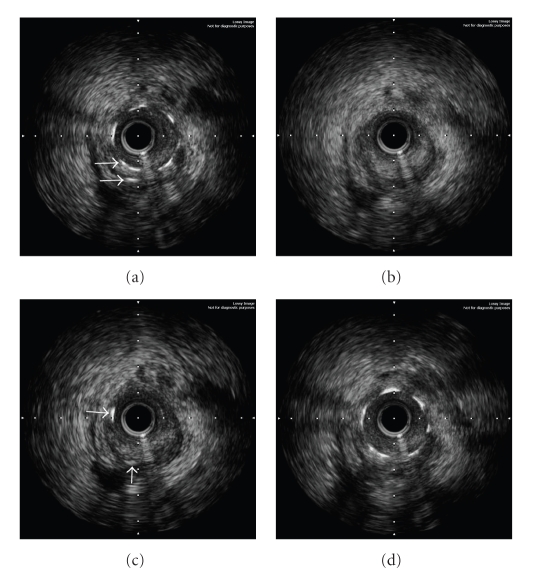
Successive IVUS images of the LAD artery from proximal (a) to distal part (d) of the fractured stent are shown after the occlusive thrombus was mechanically removed. (a) Complete, transverse fracture is seen with a spiral distortion of stent struts (parallel arrows). (b) Stent gap with totally missing struts. (c) Re-appearance of a few struts (orthogonal arrows). (d) Intact distal part of the stent.

Following the hospital discharge, the patient was instructed to take daily aspirin 100 mg for life, clopidogrel 75 mg for one year, bisoprolol 2.5 mg, ramipril 2.5 mg, and atorvastatin 10 mg. She was doing well until 280 days later when she was awakened early in the morning by the intensive chest pain accompanied by sweating and nausea. At the ED, her heart rate was 60/min, blood pressure 90/60 mm Hg, respiratory rate 18/min, and there were no pulmonary rales. The ECG demonstrated significant ST-segment re-elevations in leads V_3–6_. She received similar medication as previously, and was transferred to our catheterization laboratory again. The repeat angiography showed a thrombotic occlusion of the implanted stent (Figure 1(c)). An EBU 3.75 F6 Launcher guiding catheter (Medtronic, Minneapolis, Minn, USA) was used to engage the left main coronary artery, a 0.014” BMW guide wire (Abbot, Redwood City, Calif, USA) crossed the stent struts easily, and a 2.0 × 20 mm Mercury balloon (Abbot, Redwood City, Calif, USA) was gently inflated at 6 At to reopen the LAD. At this point, the IVUS using the Atlantis 40 MHz transducer (Boston Scientific, Maple Grove, Minn, USA) with a motorized pullback at 0.5 mm/s was performed to elucidate the mechanisms of the ST. The proximal LAD showed a positive vessel wall remodeling with maximum diameters within the external elastic membrane (EEM) at both stent borders of 4.5 mm and 3.5 mm, respectively (Figures 2(a), 2(b)). The vessel lumen, however, was encroached by a huge atherosclerotic plaque that comprised 59% to 74% of the measured area within the EEM and extended considerably beyond the outer stent margins. Near the distal part of the implanted stent, a severe stent fracture (SF) was found with complete strut separation over a wide distance (Figures 3(a)–3(d)). The cross-sectional lumen area (CSA) at the site with missing struts was only 2.1 mm^2^. The decision was made to enlarge the stent with the balloon angioplasty. The noncompliant Mercury NC 3.0 × 20 mm balloon (Abbot, Redwood City, Calif, USA) was inflated successively across the stented segment up to 20 At with the resulting CSA of ≥ 6.5 mm^2^. The final angiographic result appeared excellent (Figure 2(d)) and, therefore, the PCI was completed without additional stent placement.

The patient was then admitted to the CCU where tests addressing the antiaggregatory platelet function were carried out. The point-of-care testing using VerifyNow (Accumetrics, San Diego, Calif, USA) demonstrated a good antiaggregatory effect of aspirin with the ARU of 390 (normally, 620–672) and, on the contrary, a poor response to clopidogrel with the PRU of 245 (normally, 194–418) and only 10% inhibition, suggesting the clopidogrel resistance. The daily dose of clopidogrel was raised to 150 mg for the next year.

The further hospital course was uneventful. She experienced no additional adverse events until her outpatient visit 6 months later.

## 3. Discussion

We have described the case of the young patient in whom the SES was implanted on the culprit LAD at primary PCI for the acute MI. Nine month later she suffered from the re-MI due to the late ST. Several factors were found that may have contributed to the ST: (1) suboptimal PCI technique, (2) SF, and (3) clopidogrel resistance.

A rigorous PCI technique is a crucial measure to prevent the ST in the DESs. It is recommended to predilate the lesion carefully with a balloon shorter than planned stent length, to achieve a stent-to-artery ratio of 1.1 : 1 at nominal pressure, to cover the entire lesion with the stent including portions injured by balloon inflations, to expand the stent with high pressure inflations using a short noncompliant balloon, and to obtain a residual stenosis of <10% or CSA ≥ 5.0 mm^2^ [2, 3]. In our case, several procedural steps should be criticized. First, the lesion was pre-dilated with the balloon longer than the chosen stent. Next, rather a small (i.e., axial geographic miss) and short stent (i.e., longitudinal geographic miss) was implanted leaving a huge portion of the atherosclerotic plaque outside the stent boundaries. If the IVUS had been performed at initial PCI, we would have taken the advantage of the positive vessel remodeling for a more aggressive media-to-media stent sizing. Finally, the SES in our patient was not properly expanded as we relied on a seemingly good angiographic result.

In our patient, a wide displacement of the fractured stent fragments accompanied by the luminal collapse was discovered at repeat catheterization. The SF has been found in 1.7–18.6% of all implantations 2–699 days after the index PCI [4, 5]. Stents prone to break are often overexpanded and overlapped, those placed into tortuous arteries with angulated segments and diffuse atherosclerosis, in the RCA, saphenous vein grafts, and myocardial bridges. Target lesion in our case, located in the diffusely diseased and angulated LAD, was certainly not well prepared for the stenting. SESs, in particular, are supposed to rupture more readily since their potent antiproliferative ability may leave stent struts unsupported by the neointimal tissue [6].

Platelets are believed to play a pivotal role in the development of thrombosis following plaque rupture occurring spontaneously or at PCI. Results from the CREST [7] and RECLOSE trials [8] have clearly demonstrated that high posttreatment platelet reactivity and incomplete P2Y12 receptor inhibition are important risk factors for the ST. Recently, Price and coworkers [9] pointed out that patients with post-treatment PRU greater than the cutoff value had significantly higher rates of cardiovascular death and ST (4.6% versus 0% in controls, *P* = .004). Likewise, the PRU measured in our patient indicated a poor antiaggregatory response to clopidogrel and, as a result, the failure of the drug to prevent thrombotic events after the stenting.

In treating our patient, we had to address two major issues, stent obstruction and clopidogrel resistance. Using a plain balloon angioplasty, we achieved an acceptable vessel lumen with the CSA well over 5.0 mm^2^ without inserting another metallic stimulus into the patient's thrombotic environment. On top of the mechanical intervention, we doubled empirically the dose of clopidogrel for the next year. Antiplatelet efficacy of increased clopidogrel dosage was not evaluated after dose increase; however, lack of clinical events after dose increase suggested better antiplatelet effect of clopidogrel.

Our patient had a very premature coronary artery disease. While her risk factors might explain the early onset of the disease, presence of hypercoagulable state was not evaluated and could have contributed to her MI on both occasions. Several physiologic and pathologic conditions, such as pregnancy, oral contraceptives, homocystein, antiphospholipid antibodies, antithrombin deficiency, low protein C and S, and some genetic mutations (e.g., factor V Leiden) are often associated with a hypercoagulable state promoting thrombotic events [10–12]. However, the elevation of homocystein during MI may be related to an increase in the acute-phase reactant proteins and the measurements should be deferred for at least 6 weeks to determine the true baseline level [11]. Finally, it remains unclear why many subjects who are positive for antiphospholipid antibodies do not develop thrombosis. Although all those patients showed the evidence of endothelial activation, only platelet activity differed between thrombotic and nonthrombotic cases predicting the ineffectiveness of the additional anticoagulant therapy [12].
